# The next‐generation K‐means algorithm

**DOI:** 10.1002/sam.11379

**Published:** 2018-05-11

**Authors:** Eugene Demidenko

**Affiliations:** ^1^ Department of Biomedical Data Science and Department of Mathematics Dartmouth College Hanover New Hampshire

**Keywords:** clusterwise regression, hard classification, K‐medians, maximum likelihood, multilevel data, robust clustering, SigClust

## Abstract

Typically, when referring to a model‐based classification, the mixture distribution approach is understood. In contrast, we revive the hard‐classification model‐based approach developed by Banfield and Raftery (1993) for which K‐means is equivalent to the maximum likelihood (ML) estimation. The next‐generation K‐means algorithm does not end after the classification is achieved, but moves forward to answer the following fundamental questions: Are there clusters, how many clusters are there, what are the statistical properties of the estimated means and index sets, what is the distribution of the coefficients in the clusterwise regression, and how to classify multilevel data? The statistical model‐based approach for the K‐means algorithm is the key, because it allows statistical simulations and studying the properties of classification following the track of the classical statistics. This paper illustrates the application of the ML classification to testing the no‐clusters hypothesis, to studying various methods for selection of the number of clusters using simulations, robust clustering using Laplace distribution, studying properties of the coefficients in clusterwise regression, and finally to multilevel data by marrying the variance components model with K‐means.

## 1 INTRODUCTION

*K*‐means is the most popular clustering algorithm. A review of the technique is outside the scope of the present work—we refer the reader to a highly cited paper by Jain 20 for a general discussion.

Typically, *K*‐means is referred to as a hard classification clustering technique because the answer to whether an observation belongs to a cluster is either yes or no. In contrast, another popular classification algorithm based on a mixture (in most instances a Gaussian mixture) distribution is a soft classification technique because the answer on cluster membership is expressed in terms of a probability. An advantage of the mixture distribution approach is that the membership indicator is a continuous parameter (probability) and therefore smooth optimization methodology applies so that maximization of the likelihood function can be effectively achieved by the expectation‐maximization (EM) algorithm 26. An attractive feature of the Gaussian mixture is that it is a model‐based classification approach; therefore, traditional likelihood‐based methodologies, such as hypothesis testing or the AIC/BIC criteria, can be employed to facilitate testing of the components or to select the number of clusters. It is well forgotten that the *K*‐means also can be viewed as a model‐based approach with minimization of the total within sum of squares equivalent to the maximum likelihood (ML). However, unlike the mixture distribution approach, the classical ML theory fails here because (1) the number of parameters, as the partition index sets, exponentially increases with *n* and *K* (typically referred to as an HP‐hard problem); (2) parameters as index sets are integers (discrete) and therefore the Wald and likelihood ratio tests do not apply (the parameter value must be an inner point of the parameter space); and (3) the AIC/BIC criteria are not applicable because of the discontinuity of the parameter space.

Statistical model‐based hard classification was popularized and developed by Banfield and Raftery 2, although they do not mention the term “*K*‐means algorithm.” Remarkably, not much has been done in terms of developing and extending the model‐based *K*‐means algorithm since then. Perhaps the most attractive feature of model‐based cluster analysis, compared to a method‐based approach, is that data can be generated according to the model, and the statistical properties of clusterization can be studied via simulations.

The goal of the present work is to revive and extend the ideas presented by Banfield and Raftery by viewing the *K*‐means algorithm as the ML technique in several directions: (1) testing the presence of clusters and computing the *p*‐value; (2) identification of the number of clusters; (3) viewing the *K*‐medians algorithm as the ML based on the Laplace distribution; (4) developing a semisupervised *K*‐means algorithm in the case of a priori information; (5) developing the clusterwise *K*‐means regression; and, finally, (6) extension of the *K*‐means algorithm to clustering of multilevel data in the presence of replicates. However, it is not the goal of this work to develop new numerical algorithms. Instead, our hard classification procedures are reduced to the repeated application of the existing and efficient Hartigan‐Wong 17 algorithm. In the present work, only the spherical Gaussian distribution is assumed; an extension to the case when observations are heteroscedastic or correlated, as studied by Banfield and Raftery 2, can be carried out along the lines of the spherical case and is a topic of future research.

## 2 SPHERICAL GAUSSIAN DISTRIBUTION

In this section, we consider the simplest model‐based hard classification problem leading to the *K*‐means algorithm. It is assumed that *n* independently distributed observation vectors **x**_1_, **x**_2_, …, **x**_*n*_ ∈ *R* ^*m*^ are independent and belong to *K* groups specified by the index sets *C*_1_, *C*_2_, …, *C*_*K*_. These index sets partition the set {1, 2, …., *n*} so that $\cup_{k=1}^{K}C_{k}=\left\{1,,,2,,,\ldots ,,,n\right\}$ and *C*_*k*_ ∩ *C*_*l*_ = ∅ for *k* ≠ *l*. The distribution of observations from each cluster is identical to the common variance. Moreover, it is assumed that the distribution is spherical Gaussian:

(1) $$x_{i}∼N\left(\mu_{k},\sigma^{2}I_{m}\right),\;i\in C_{k}.$$

The parameters to estimate are the means {**μ**_*k*_, *k* = 1, 2, …, *K*}, the common variance *σ* ^2^, and, most importantly, the index sets (*C*_1_, *C*_2_, …, *C*_*K*_). The twice‐negative log‐likelihood function takes the form

(2) $$\mathrm{mn}\ln\sigma^{2}+\sigma^{-2}\sum_{k=1}^{K}\sum_{i\in C_{k}}{\left\|x_{i}-\mu_{k}\right\|}^{2}.$$

Differentiating with respect to **μ**_*k*_, we find that, given the index sets, the ML estimator is

$${\bar{x}}_{k}=\frac{1}{n_{k}}\sum_{i\in C_{k}}x_{i},$$

where *n*_*k*_ is the number of elements in the cluster *k*. Differentiating (2) with respect to *σ* ^2^, we find that the ML estimation is equivalent to the minimum of the total within sum of squares:

(3) $$S_{K}=\min_{C_{1},\ldots ,C_{K}}\sum_{k=1}^{K}\sum_{i\in C_{k}}{\left\|x_{i}-{\bar{x}}_{k}\right\|}^{2}.$$

Thus, ML with a spherical Gaussian distribution is equivalent to the traditional *K*‐means algorithm. The minimization of criterion (3) is not trivial and may have multiple minima, so several starting points may be used to confirm that the global minimum is found. An ML estimate of the variance is ${\hat{\sigma }}^{2}={\left(\mathrm{mn}\right)}^{-1}S_{K},$ as follows from (2).

An immediate implication of the fact that the *K*‐means algorithm solves the ML problem is an obvious but sometimes ignored consequence that the *K*‐means algorithm is applicable only to normally distributed data with equal variance. Consequently, the *K*‐means algorithm is not justified for uniformly distributed data or when vector components are measured on different scales and therefore have different variances. One might suggest normalizing the original data by subtracting the gross mean and dividing by the standard deviation, but such normalization would be suboptimal because the variance should be computed around the mean in each cluster, not around the gross mean.

### 2.1 Testing the presence of clusters

**Figure 1:**
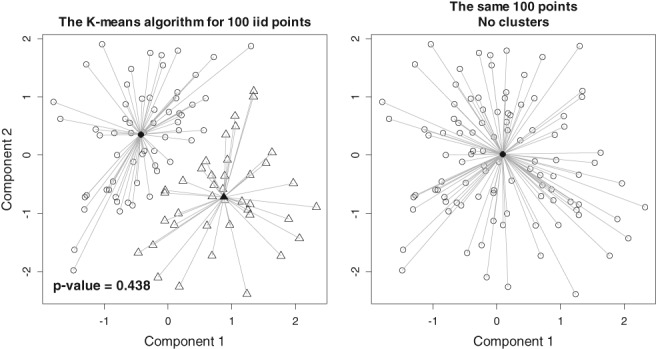
The K‐means algorithm with K = 2 for a sample of 100 random points from the same bivariate normal distribution with zero mean and unit variance. A wrong clusterization is shown in the right plot (the same points)!

A fundamental question is: Are there clusters? A false clusterization is illustrated in Figure 1. The *K*‐means algorithm with 2 clusters (*K* = 2) is applied to *n* = 100 points generated from the same normal distribution with zero mean, unit variance, and zero correlation (spherical Gaussian distribution). The *K*‐means algorithm divides these points into 2 clusters, but in fact there are no clusters because points are generated from the same distribution. Visualization may be deceiving. Needless to say, the absence of clusters becomes even more difficult to detect for higher dimensions (*m* > 2).

We aim to test whether points {**x**_*i*_, *i* = 1, 2, …, *n*} belong to the same normal population—that is, there are no clusters. This hypothesis will be referred to as the *no‐clusters* hypothesis. Clustering tendency bothered mathematicians from the very beginning 42, but most of the work has been done in an asymptotic setup when *n* → ∞ . We mention just a sample of authors: Pollard 27, Bryant and Williamson 7, Bock 5, and Jain and Dubes 19. Unlike previous research, we want to compute the *p*‐value for testing the no‐clusters hypothesis for small *n*. The idea is to use the established MANOVA test statistic when the index sets are known. The key observation is that, for the *K*‐means algorithm, the index sets are unknown and subject to estimation. Therefore, a distribution, such as the *F*‐distribution, does not hold. This distribution will be derived via simulations; see also refs. 22, 25, 30.

We say that there are no clusters if the null hypothesis *H*_0_: **μ**_1_ = **μ**_2_ = ⋯ = **μ**_*K*_ is not rejected with the given Type I error *α* (typically, *α* = 0.05). If the index sets *C*_*k*_ were known, the traditional exact *F*‐test or approximate likelihood ratio (LR) MANOVA test could be applied, Anderson 1. These are based on the total and within‐cluster sums of squares

(4) $$S_{1}=\sum_{i=1}^{n}{\left\|x_{i}-\bar{x}\right\|}^{2},\;S_{K}=\min_{C_{1},\ldots ,C_{K}}\sum_{k=1}^{K}\sum_{i\in C_{k}}{\left\|x_{i}-{\bar{x}}_{k}\right\|}^{2},$$

respectively. When the index sets are unknown and estimated, as in the *K*‐means algorithm, the distribution of classical statistics does not hold, so the classical MANOVA does not apply.

To compute the *p*‐value for the no‐clusters hypothesis when the index sets are unknown, we need to estimate the cumulative distribution function (cdf) of statistics under the null hypothesis: that is, when $x_{i}∼N\left(\mu ,\sigma^{2}I\right),$ *i* = 1, 2, …, *n*. We could use either the *F*‐statistic, (*S*_1_ − *S*_*K*_)/*S*_*K*_, or the likelihood ratio test, log(*S*_1_/*S*_*K*_), but the *p*‐value does not change upon any strictly increasing transformation, so it suffices to find the cdf of the ratio

(5) $$r=\frac{S_{1}}{S_{K}}.$$

The advantage of the statistic (5) is that its distribution, under the null hypothesis, does not depend on **μ** and *σ* ^2^. Indeed, simple algebra proves that

$$r=\frac{S_{1}/\sigma^{2}}{S_{K}/\sigma^{2}}=\frac{S_{1z}}{S_{\mathrm{Kz}}},$$

where

$$S_{1z}=\sum_{i=1}^{n}{\left\|z_{i}-\bar{z}\right\|}^{2},\;S_{\mathrm{Kz}}=\min_{C_{1},\ldots ,C_{K}}\sum_{k=1}^{K}\sum_{i\in C_{k}}{\left\|z_{i}-{\bar{z}}_{k}\right\|}^{2},$$

and $z_{i}∼N\left(0,I\right)$.

Finally, the method of computing the *p*‐value for the no‐clusters null hypothesis versus the alternative that the number of clusters is *K* is as follows: Let the *K*‐means algorithm for the data at hand {**x**_*i*_, *i* = 1, 2, …, *n*} produce r_*_ as the ratio of 2 sums of squares (5). Carry out a fairly large number of simulations *N*, say *N* = 1000, to obtain the empirical cdf of *r*: For each simulation, (1) generate $\left\{z_{1},z_{2},\ldots ,z_{n}\right\}∼N\left(0,I\right)$, (2) run *K*‐means, and (3) compute the total sum of squares *S*_1*z*_, the within sum of squares from the *K*‐means, *S*_*Kz*_, and *r* = *S*_1*z*_/*S*_*Kz*_. It took about 2 s for the data depicted in Figure 1 to do simulations in R on a regular desktop using 10 random initialization starts. Then, the *p*‐value is the proportion of simulations in which *r* > r_*_. If there were clusters, then r_*_ would be greater than the typical *r* under the null hypothesis (no clusters). Typically, we say that the null hypothesis is rejected if the proportion (*p*‐value) is < 0.05. The *p*‐value for the configuration of points depicted in Figure 1 is 0.438. This means that the no‐clusters hypothesis cannot be rejected. If the number of simulations *N* is fairly large, the *p*‐value is computed with precision of order 1/*N*.

**Figure 2:**
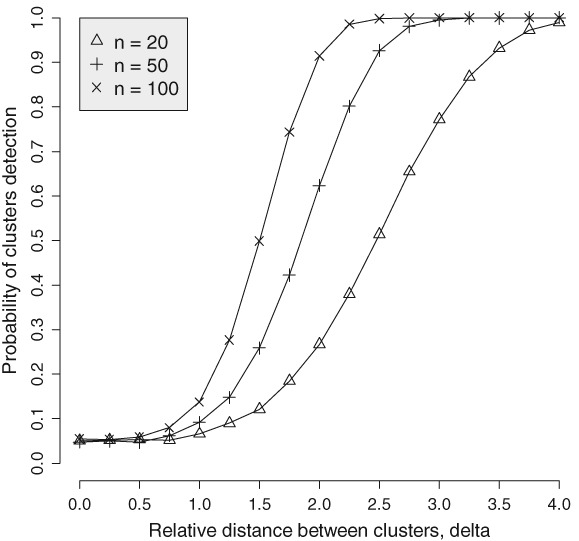
Three power functions for detection of 2 clusters with the delta on the x‐axis (K = 2 and K = 2)

The typical threshold for the *p*‐value, 0.05, specifies Type I error (the alpha error): the probability of concluding that there are several clusters when in fact there are no clusters. Type II error (the beta error) is the probability of concluding that there are no clusters when in fact there are clusters. Usually, we compute the power function as complement to the beta error, that is, the probability of rejecting clusters when in fact there are clusters. Of course, the power function depends on how separated the clusters are. For example, in the case of 2 clusters, the power function depends on the Mahalanobis distance, ||**μ**_1_ − **μ**_2_||/*σ* = *δ*. When *δ* = 0, the power function turns into Type I error *α*; when *δ* → ∞, the power function approaches 1. The power function tells how different the centers of the clusters, adjusted for *σ*, must be to claim that there are 2 clusters. An example of the power function for cluster detection is shown in Figure 2 for different *n* and *m* = 2. More points produce a higher probability of cluster detection. With 20 points, one needs to have the distance *δ* ≈ 3 to be able to detect the cluster configuration with probability ∼80%.

### 2.2 How many clusters: the broken‐line algorithm

“What is *K*?” is the paramount question of the *K*‐means algorithm, Hastie et al. 18. There is a rich body of literature on the topic, and it is not the objective of the present work to review available methods for choosing the number of clusters in the *K*‐means algorithm. Instead, we develop a new *broken‐line* algorithm and compare its performance via simulations against 27 other algorithms of *K* determination computed by the R function NbClust based on the statistical model (1); see the next section.

Our broken‐line algorithm is an elaboration of the well‐known and loosely defined elbow method: (1) Plot the log total within sum of squares, *S*_*K*_, against *K* for a sequence of values *K* = 1, 2, …, *K*_max_, and (2) chose *K* at the elbow of the curve, that is, where the line exhibits a change of slope. Although this method is intuitively appealing, there is no formal rule to define the elbow. We facilitate the determination of *K* by plotting ln*S*_*K*_ and identifying *K* where the rate of decrease of ln*S*_*K*_ (the slope) changes. Precisely, the broken‐line algorithm is as follows: Fit 2 linear regressions using 2 segments of the data, {*S*_1_, *S*_2_, .., *S*_*K*_} and $\left\{S_{K+1},S_{K+2},..,S_{K_{\max}}\right\}$, and compute the total residual sum of squares for *K* = 2, 3, …, *K*_max_ − 2. The optimal *K* is where the sum of squares takes a minimum.

**Figure 3:**
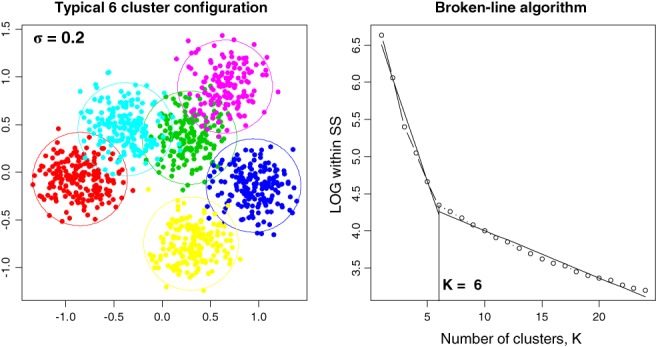
An illustration of the broken‐line algorithm for the determination of the number of clusters

This algorithm is illustrated in Figure 3. In the left plot, 6 clusters are simulated according to model (1) using *σ* = 0.2 with about 150 points in each cluster. The circles depict the 95% confidence region with centers at the true mean and radius $\sigma \sqrt{\chi^{-2}\left(0.95,,,2\right)},$ where *χ* ^−2^ (0.95, 2) is the 0.95th quantile of the chi‐distribution with 2 degrees of freedom. In the right plot, we run 24 kmeans algorithms, letting *K* = 1, 2, …, 24 (=*K*_max_) and plot ln*S*_*K*_ against *K*. Then we run 23 × 2 = 46 linear fits and find the pair that produces the minimum total residual sum of squares. The minimum occurs at *K* = 6. Note that plotting *S*_*K*_ against *K*, as usually recommended, does not detect the change in slope—the log scale is crucial.

Although no theoretical justification for using lnS_K_ is offered in this work yet, the link back to the log‐likelihood (2) can be easily traced. Indeed, the minimum twice‐negative log‐likelihood is mn[lnS_K_ − ln(mn) + 1]. Since m and n are K‐independent, the optimal log‐likelihood solely depends on lnS_K_, which is the prime metric in the famous Neyman‐Pearson lemma for hypothesis testing, Lehmann and Romano 24.

EXAMPLE 1 Human tumor microarray data. Hastie et al. 18, p. 512 provide an example of the K‐means algorithm with n = 64 human tumors to be classified in groups using 6830 gene microarray expression data. As stated in the book, “…there is no clear indication” on the number of clusters; they use K = 3. The identification of the number of clusters in this example based on our broken‐line algorithm is depicted in the left plot of Figure 6 . Although visual identification of the elbow is indeed difficult even on the log scale, the rate of the drop changes at K = 5 (the gap statistic identified 2 clusters).

#### 2.2.1 Comparison with other methods using simulations

**Figure 4:**
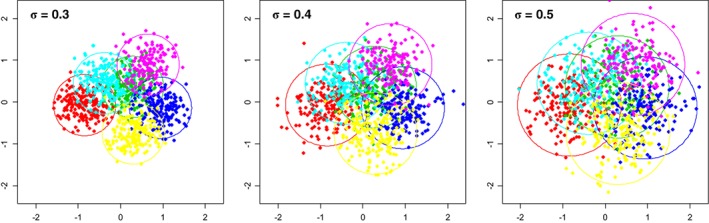
Typical point configurations for σ = 0.3, 0.4, and σ = 0.5

We use the package NbClustin R to compare our broken‐line algorithm against 27 other methods previously reported in the literature over the years, including a popular gap statistic method by Tibshirani et al. 36. We simulate six clusters according to model (1) with σ = 0.2, 0.3, 0.4, and 0.5, with typical configurations shown in Figure 4; a typical configuration for σ = 0.2 is depicted in Figure 3.

The best 6 methods of *K* determination are presented in Table 1; we do not report the results of classification on the other 21 methods, including gap statistic, because they are worse in terms of the deviation of the identified number clusters from *K* = 6. For each method, we compute the mean of the identified *K* across simulations, $\bar{K},$ to evaluate the bias; the standing is determined by the absolute deviation of averages from 6 across *σ* (the last column). It is understandable that, when *σ* increases (clusters are getting wider), the methods tend to find fewer clusters. The superiority of the broken‐line algorithm is obvious.

**Figure 5:**
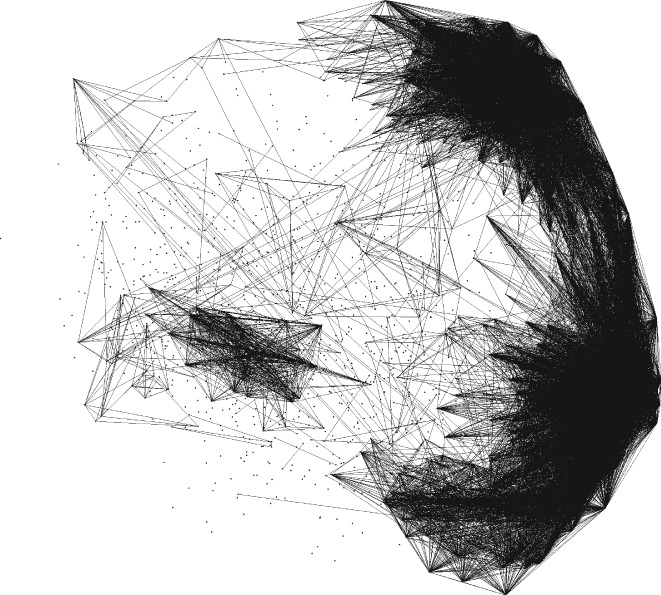
PCA of n = 1500 ovarian tumor genes. Points are connected if the coefficient of determination is >0.47. Four clusters can be recognized—our broken‐line algorithm identifies 4 clusters as well, see the right plot in Figure 6

**Figure 6:**
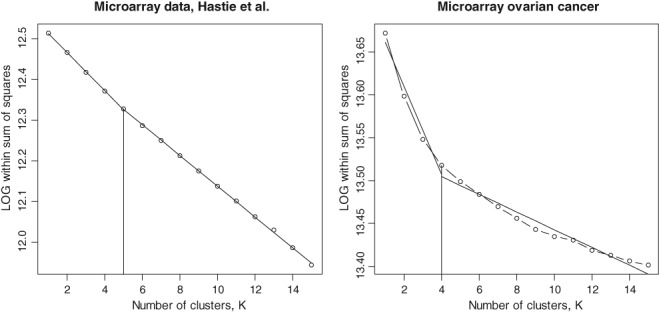
The broken‐line algorithm for 2 sets of microarray data. Left: The number of clusters in the microarray of 64 human tumors 18, K = 5. Right: The number of clusters (subtypes) of the ovarian cancer using m = 1500 microarrays among n = 489 individuals, K = 4

EXAMPLE 2 *Classification of ovarian cancer microarrays*. The identification of latent clusters of genes of ovarian cancer is an important problem for improving treatment outcomes 32. Considerable effort has been devoted by The Cancer Genome Atlas (TCGA) Research Network researchers to carry out microarray experiments to identify clusters of genes that could better classify the disease with a possibility of gene therapy 33. However, the number of gene clusters is still an open question. Several researchers hypothesize that the number of clusters of genes should be equal to the number of clinically supported ovarian tumor subtypes: serous, mucinous, endometrioid, and clear cell 39. Here we use the gene expressions data of the *n* = 1500 most representative genes from *m* = 489 ovary tumors 12. Figure 5 depicts the principal component analysis (PCA) of 1500 points from *R* ^489^ points representing genes that are connected if the coefficient of determination (squared Pearson correlation coefficient) is greater than 0.3. Four clusters can be recognized—connecting the pairs of points by a segment improves the clusters' visibility. The plot of ln*S*_*K*_ against *K* is shown at right in Figure 6. The regression lines for [1, 2, 3, 4] and [5, 6, …, 15] yield minimum residual sum of squares: the broken‐line algorithm confirms that the number of clusters of genes is 4.

**Table 1:** Comparison of 6 methods of estimation of the number of clusters via simulations

			*σ*				Mean
Rank	Method	Reference	0.2	0.3	0.4	0.5	$|\bar{K}-6|$
1	Broken‐line	Present work	6.0	5.1	5.6	6.0	0.32
2	CH	Calinski and Harabasz 8	6.1	6.1	3.0	3.0	1.55
3	Silhouettes	Rousseeuw 28	5.8	5.8	3.0	3.0	1.60
4	KL	Krzanowski and Lai 23	3.9	3.9	7.8	4.5	1.88
5	SDindex	Halkidi et al. 16	5.0	5.0	3.0	3.4	1.90
6	CCC	Sarle 34	6.1	6.1	2.0	2.0	2.05

## 3 K‐MEDIANS CLUSTERING ALGORITHM

In reality, observations may contain outliers or even observations that do not belong to either cluster. In this section, we suggest a statistical model for the *K*‐medians clustering algorithm. The *K*‐medians is a well‐known robust version of hard clustering—we will derive this algorithm via the method of ML using the multivariate Laplace distribution. Although the application of Laplace distribution to mixture distribution and fuzzy clustering is known 3, 6, 9, 13, 29, 34, 37, we are not aware of derivation of the *K*‐medians algorithm through the method of ML, but most importantly by taking full advantage of a statistical model‐based approach by (1) applying classical statistical tests to answer important questions about clusters, (2) computing the confidence region for each cluster, and, finally, (3) generating data and carrying out simulations to study statistical properties of statistical tests and estimators.

Denote with ℒ(*μ*, *θ*) the Laplace (or double‐exponential) distribution with the density *f*(*x*; *μ*, *θ*) = (2*θ*)^−1^*e*^−|*x* − *μ*|/*θ*^, where *μ* is referred to as the location parameter and *θ* is referred to as the scale parameter. It is well known that, if $x_{i}\overset{\text{iid}}{∼}ℒ\left(\mu ,\theta \right),$ then the ML estimator of *μ* is the median and solves the minimization problem $\sum_{i=1}^{n}\left|x_{i}-\mu \right|\Rightarrow \min$. This fact is the impetus for our statistical model: It is assumed that the components of the *m*‐dimensional vector **x**_*i*_ from cluster *k* are independent and identically distributed with the location parameter **μ**_*k*_ and the common scale parameter *θ* (vector observations are independent as well). Symbolically

$$x_{i}∼ℒ\left(\mu_{k},\theta I_{m}\right),\;i\in C_{k}.$$

The log‐likelihood function, up to a constant term, takes the form

$$\begin{array}{cc} & l\left(\mu_{1},\ldots ,\mu_{K},\theta ,C_{1},C_{2},\ldots ,C_{k}\right)\\ & =-\left(\mathrm{mn}\ln\theta +\theta^{-1}\sum_{k=1}^{K}\sum_{i\in C_{k}}\left|x_{i}-\mu_{k}\right|\right). \end{array}$$

Commonly, |**x**_*i*_ − **μ**_*k*_| refers to the *L*_1_‐norm or Manhattan distance between the observation vector **x**_*i*_ and the respective center **μ**_*k*_. Obviously, the maximum of *l* occurs when

$$\min_{C_{1},\ldots ,C_{K}}\sum_{k=1}^{K}\sum_{i\in C_{k}}\left|x_{i}-{\tilde{x}}_{k}\right|,$$

where ${\tilde{x}}_{k}$ is the *m* × 1 median vector in cluster *k*. This implies that the method of ML with the Laplace distribution is equivalent to the *K*‐medians algorithm.

Now we illustrate how the no‐clusters test can be generalized to the *K*‐medians: As before, the test statistic is the ratio (5), but now

$$S_{1}=\sum_{i=1}^{n}\left|x_{i}-\tilde{x}\right|,\;S_{K}=\min_{C_{1},\ldots ,C_{K}}\sum_{k=1}^{K}\sum_{i\in C_{k}}\left|x_{i}-{\tilde{x}}_{k}\right|,$$

where $\tilde{x}$ is the overall median vector assuming no clusters. It is easy to see that, similar to the Gaussian case, the ratio *r* = *S*_1_/*S*_*K*_ does not depend on either **μ** or *θ*. This means that we can estimate the cdf of *r* from simulations using $z_{i}\overset{\mathrm{iid}}{∼}ℒ\left(0,I_{m}\right)$ instead of **x**_*i*_. Then the *p*‐value for testing the null hypothesis that there are no clusters is the *α*th quantile of the empirical cdf (typically we use *α* = 0.05).

**Figure 7:**
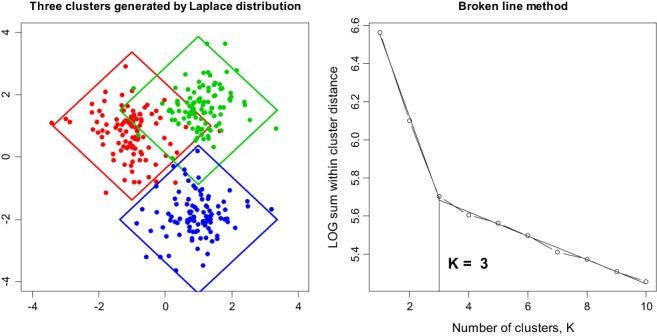
Three clusters are generated using the Laplace distribution with the 45° rotated squares as the 95% confidence regions. The broken‐line algorithm correctly identifies K = 3

The broken‐line algorithm for selection of *K* generalizes to *K*‐medians in a straightforward manner and is illustrated in Figure 7, where observations from 3 clusters are generated according to the Laplace distribution. We used the R function cclust of the package flexclust to run the *K*‐medians algorithm.

The (1 − *α*)th confidence region for each cluster is constructed using the fact that, if $x_{i}\overset{\text{iid}}{∼}ℒ\left(\mu ,\theta \right),$ then $2\theta^{-1}\sum_{i=1}^{n}\left|x_{i}-\mu \right|∼\chi^{2}\left(2n\right).$ In particular, for *m* = 2, as in Figure 7, the confidence region for the *k*th cluster is the 45° rotated square (rhombus) and is defined by the equation |*x*_1_ − *μ*_1_| + |*x*_1_ − *μ*_2_| = 0.5*θχ*^−2^(0.95, 4), where *χ* ^−2^(0.95, 4) is the 0.95th quantile of the chi‐distribution with 4 degrees of freedom (the left plot). The right plot shows that the broken‐line algorithm correctly determines the number of clusters.

## 4 SEMISUPERVISED K‐MEANS ALGORITHM

A common critique of cluster analysis is that it does not make a connection between cluster and group. The labeling and interpretation is up to the user because cluster analysis is an unsupervised classification technique. Sometimes, one has an additional set of observations from some clusters to put the labels right. Several authors have suggested variants of the *K*‐means algorithm to account for observations with known labels/groups. For example, Wagstaff et al. 41 and Basu et al. 4 suggested improving the *K*‐means algorithm starting from seeding generated by the label‐known (known membership) observations or do clustering in the restricted sense, so that all observations with known membership belong to the same cluster. However, unlike previous authors, we suggest the incorporation of a priori knowledge using a model‐based approach.

We use the following example to illustrate the *K*‐means when the cluster membership of some observations is known (these observations will be referred to as supervised observations). That is why this version will be called the semisupervised *K*‐means algorithm. The following example clarifies the concept.

**Figure 8:**
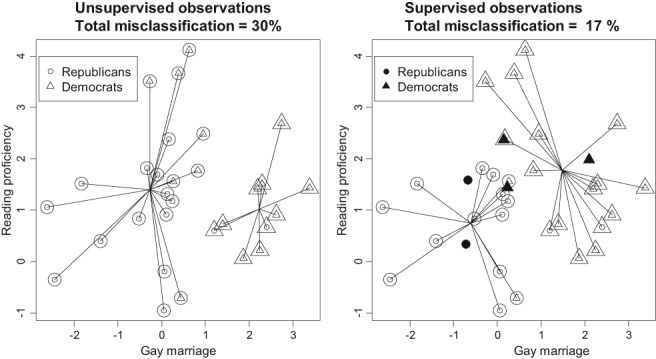
K‐means with and without a priori classification. The addition of five individuals with known party (solid symbols) improves the discrimination

EXAMPLE 3 *Political party classification*. We want to use reading proficiency and attitude toward gay marriage to identify whether the individual is a Democrat or a Republican. Thirty people were tested for reading proficiency, and the question was asked about their opinion on gay marriage. In addition, each person reported his/her political party, Republican (circle) or Democrat (triangle); the measurements were transformed into a scoring system where 0 means national average; see Figure 8. The party membership information was not used for classification but for computing the misclassification error. The standard *K*‐means algorithm was applied to classify the 30 people into 2 groups. Small circles and triangles indicate the true party membership, and large circles and triangles indicate the *K*‐means classification accordingly (an individual is misclassified if the symbols are different). As follows from the left plot, one Republican was mistakenly classified as a Democrat, but there are many more Democrats misclassified as Republicans. Overall, 30% of individuals were misclassified. In the right plot, the same points are used, but, in addition, we have 5 individuals (supervised observations) with known party, marked as solid symbols. The question is: How to incorporate the supervised observations into the classification algorithm and what is the appropriate statistical model?

Now we describe the statistical model for the hard classification that incorporates observations with known clusters. As in the regular *K*‐means model, it is assumed that unsupervised observations follow the assumption

$$x_{i}∼N\left(\mu_{i},\sigma^{2}\right),\;i=1,2,\ldots ,n,$$

where **μ**_*i*_ = **μ**_*k*_ for *i* ∈ *C*_*k*_, *k* = 1, 2, …, *K*. In addition to these *n* points, we have *p*_*k*_ supervised observations for the *k*th cluster. Note that *p*_*k*_ ≥ 0, and in a special case when *p*_*k*_ = 0 for all *k* = 1, …, *K*, we come to the standard *K*‐means model. The twice negative log‐likelihood function, up to a constant term, is

$$\begin{array}{cc} & m\left(n+P\right)\ln\sigma^{2}\\ & +\sigma^{-2}\left(\sum_{k=1}^{K}\sum_{i\in C_{k}}{\left\|x_{i}-\mu_{k}\right\|}^{2}+\sum_{k=1}^{K}\sum_{j=1}^{p_{k}}{\left\|y_{\mathrm{kj}}-\mu_{k}\right\|}^{2}\right). \end{array}$$

Equating the derivative with respect to *σ* ^2^ to zero, we reduce the ML estimation to the following minimization problem:

$$\sum_{k=1}^{K}\sum_{i\in C_{k}}{\left\|x_{i}-\mu_{k}\right\|}^{2}+\sum_{k=1}^{K}\sum_{j=1}^{p_{k}}{\left\|y_{\mathrm{kj}}-\mu_{k}\right\|}^{2}.$$

If the index sets {*C*_*k*_} are held fixed, differentiation with respect to **μ**_*k*_ leads to the solution

$$\begin{array}{cc} {\hat{\mu }}_{k} & =\frac{1}{n_{k}+p_{k}}\left(\sum_{i\in C_{k}}x_{i}+\sum_{j=1}^{p_{k}}y_{\mathrm{kj}}\right)=\frac{n_{k}}{n_{k}+p_{k}}{\bar{x}}_{k}+\frac{p_{k}}{n_{k}+p_{k}}{\bar{y}}_{k}\\ & =-\frac{p_{k}}{n_{k}+p_{k}}{\bar{x}}_{k}+\frac{p_{k}}{n_{k}+p_{k}}{\bar{y}}_{k}+{\bar{x}}_{k}=\frac{p_{k}}{n_{k}+p_{k}}\left({\bar{y}}_{k}-{\bar{x}}_{k}\right)+{\bar{x}}_{k}. \end{array}$$

We use this derivation to solve the ML hard classification via the repeated *K*‐means algorithm:

1. Apply the regular *K*‐means to *n* unsupervised observations {**x**_*i*_}.
2. Adjust

(6) $$x_{*i}=x_{i}-\frac{p_{k}}{n_{k}+p_{k}}\left({\bar{x}}_{k}-{\bar{y}}_{k}\right)$$

and apply the *K*‐means to $n+\sum_{k=1}^{K}p_{k}$ points {x_*i_} iterating until convergence.

To understand the adjustments (6), find the center of the *k*th cluster for observations {x_*i_}:

$$\frac{1}{n_{k}}\sum_{i\in C_{k}}x_{*i}={\bar{x}}_{k}-\frac{p_{k}}{n_{k}+p_{k}}\left({\bar{x}}_{k}-{\bar{y}}_{k}\right)=\frac{n_{k}}{n_{k}+p_{k}}{\bar{x}}_{k}+\frac{p_{k}}{n_{k}+p_{k}}{\bar{y}}_{k}.$$

As follows from this algebra, the adjustments (6) can be viewed as the weighted means using ${\bar{x}}_{k}$ and ${\bar{y}}_{k}$ with the weights proportional to the number of unsupervised and supervised observations in cluster *k*, respectively. Typically, it takes 1 or 2 iterations to converge.

This algorithm was applied to the above example (see the right plot of Figure 8), and it converged in 2 iterations. The addition of supervised observations improved the discrimination: the total misclassification error dropped from 30% to 17%.

## 5 CLUSTERWISE REGRESSION

Most literature takes the soft clusterization, mixture distribution, approach to linear regression, for example, Yan et al. 38. An extension of the hard classification to the linear regression model is also known and called clusterwise regression, Spath 31. In this section, we suggest a statistical model for clusterwise regression, reduce the ML estimation to repeated *K*‐means, demonstrate how the distribution of the regression coefficients can be studied via simulations, and, finally, generalize clusterwise regression to multiple dependent variables.

### 5.1 Single dependent variable

If *y*_*i*_ is the *i*th observation of the dependent variable and **x**_*i*_ is the respective *m* × 1 vector of independent (explanatory) variables, it is assumed that, within each cluster, there is its own vector of regression coefficients

$$y_{i}∼N\left(\beta_{k}^{\prime }x_{i},\sigma^{2}\right),\;i\in C_{k},\;k=1,2,\ldots ,K,$$

under a standard assumption that observations {*y*_*i*_, *i* = 1, 2, …, *n*} are independent. As in the case of regular *K*‐means, the task is not only to estimate the *Km* regression coefficients but also to identify to what cluster each observation *i* belongs: that is, to find/estimate the partition of the set {1, 2, …, *n*} into *K* nonoverlapping index sets {*C*_*k*_}. If index sets were known, the residual sum of squares within cluster *k* could be expressed using the generalized matrix inverse:

$$\min_{\beta_{k}}\sum_{i\in C_{k}}{\left(y_{i}-x_{i}^{\prime }\beta_{k}\right)}^{2}=y_{k}^{\prime }\left(I-X_{k}\%{\left(X_{k}^{\prime }X_{k}\right)}^{+}X_{k}^{\prime }\right)y_{k},$$

where **y**_*k*_ is the *n*_*k*_ × 1 vector of the dependent variable, and **X**_*k*_ is the *n*_*k*_ × *m* matrix of independent variables composed of vectors **x**_*i*_ (*n*_*k*_ is the number of observations in cluster *k*). This formula covers the cases when *n*_*k*_ < *m* or when matrix **X**_*k*_ does not have full rank. Simple algebra shows that the ML estimation reduces to the following optimization problem:

(7) $$\max_{C_{1},\ldots ,C_{K}}\sum_{k=1}^{K}y_{k}^{\prime }X_{k}{\left(X_{k}^{\prime }X_{k}\right)}^{+}X_{k}^{\prime }y_{k}.$$

This representation gives rise to another interpretation of clusterwise regression. To simplify, let us assume that matrix **X**_*k*_ has full rank. Noting that $\sigma^{2}\left(X_{k}^{\prime }X_{k}\right)={\text{cov}}_{k}$ is the covariance matrix of ${\hat{\beta }}_{k},$ rewrite

$$y_{k}^{\prime }X_{k}{\left(X_{k}^{\prime }X_{k}\right)}^{-1}X_{k}^{\prime }y_{k}={\hat{\beta }}_{k}^{\prime }\left(X_{k}^{\prime }X_{k}\right){\hat{\beta }}_{k}=\sigma^{2}{\hat{\beta }}_{k}^{\prime }{\text{cov}}_{k}^{-1}{\hat{\beta }}_{k}.$$

Thus (7) can be interpreted as the maximization of the total significance test statistic in the Wald test.

The *K*‐means regression analysis can be extended to the case when clusters share regression coefficients (supplied with the subscript 0):

(8) $$y_{i}∼N\left(\beta_{0}^{\prime }x_{0i}+\beta_{k}^{\prime }x_{i},\sigma^{2}\right),\;i\in C_{k}.$$

For example, the clusters may have the same slopes but different intercepts (see an example below).

To estimate the clusterwise regression with shared coefficients (8), the following repeated *K*‐means algorithm is proposed: (0) apply the least squares to the entire dataset and compute residuals *r*_*i*_; (1) apply the *K*‐means to residuals {*r*_*i*_} to classify them into *K* clusters (classification on the real line); (2) estimate **β**_0_ and **β**_*k*_ in each cluster separately using the dummy‐variable approach and compute new residuals, and return to step (1). Iterate until convergence. The following example illustrates the repeated *K*‐means algorithm for the clusterwise regression.

**Figure 9:**
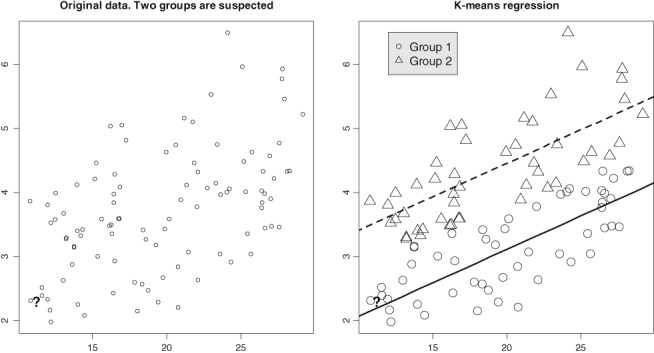
Left: The original data does not reveal 2 groups although their existence is suspected. Right: The K‐means reveals 2 groups (they have the same slope but different intercepts)

EXAMPLE 4 *Two group regressions with common slope*. Consider a simple linear regression *y*_*i*_ = *β*_0_ + *β*_1_ *x*_*i*_ + *ϵ*_*i*_, where *i* = 1, 2, …, *n* denotes the subject id. The data is suspected to combine 2 groups with different baselines—the intercepts are group‐specific, but the slope coefficient *β*_1_ is the same (the groups are unknown and subject to estimation). Specifically, we want to know what group the subject depicted with “?” belongs to (the left bottom corner); see Figure 9. The statistical model is *y*_*i*_ = *β*_01_ + *β*_1_ *x*_*i*_ + *ϵ*_*i*_ if *i* ∈ *C*_1_, and *y*_*i*_ = *β*_02_ + *β*_1_ *x*_*i*_ + *ϵ*_*i*_ if *i* ∈ *C*_2_, where *C*_1_ ∩ *C*_2_ = ∅ and *C*_1_ ∪ *C*_2_ = {1, 2, …, *n*}. We start by fitting the data at left with the least squares regression, treating the data as 1 sample. Then we compute the residuals and apply the *K*‐means algorithm to separate the residuals into 2 groups and obtain the first index set approximation, *C*_1_ and *C*_2_. Next, we introduce 2 dummy variables, *d*_1*i*_ = 1 if *i* ∈ *C*_1_ and 0 otherwise, and *d*_2*i*_ = 1 if *i* ∈ *C*_2_ and 0 otherwise (*d*_1_ and *d*_2_ are orthogonal). In the next step, we run the linear model *y*_*i*_ = *δ*_1_ *d*_1*i*_ + *δ*_2_ *d*_2*i*_ + *β*_1_ *x*_*i*_ + *ϵ*_*i*_ and obtain the residuals; we apply the *K*‐means again to obtain the next index set, *C*_1_ and *C*_2_, and iterate in this fashion while the total residual sum of squares decreases. It took 2 iterations for the data in Figure 9 to converge. The plot at right depicts the results of the clusterwise regression. To indicate the classification, we use different symbols; the regression lines are parallel because the groups have common slope. The question mark subject belongs to Group 1.

### 5.2 Multidimensional dependent variable

Here we generalize the above example to the case when the dependent variable **y** is *m*‐dimensional 40. Let **X**_*i*_ denote the known *m* × *p* matrix of explanatory variables, *i* = 1, 2, …, *n*. As before, we assume that vectors from different clusters have different means (intercepts) but the same slopes. Then the statistical model takes the form

(9) $$y_{i}∼N\left(\mu_{k}+X_{i}\nu ,\sigma^{2}I_{m}\right),\;i\in C_{k},$$

where **μ**_*k*_ is the *m* × 1 vector of cluster‐specific intercepts, and **ν** is the *p* × 1 vector of common slope coefficients. Matrix **X**_*i*_ should not contain a column of 1's (no intercept) because the model will be not identifiable otherwise—the intercepts are captured by **μ**_*k*_. It is easy to see that maximization of the log‐likelihood function turns into the minimization of

(10) $$\min_{\nu ,\mu_{k},C_{k}}\sum_{k=1}^{K}\sum_{i\in C_{k}}{\left\|y_{i}-\mu_{k}-X_{i}\nu \right\|}^{2}.$$

The following repeated *K*‐means algorithm is proposed for minimization of this criterion: (0) Estimate the intercepts and slopes treating the data as 1 cluster by stacking {**y**_*i*_} into the *nm* × 1 vector **y** and {**X**_*i*_} into the *nm* × *p* matrix **X**. To represent the vector of cluster‐specific intercepts, **μ**, let **Z = 1**_*n*_ **⊗ I**_*m*_ (stack *n* is the *m* × *m* identity matrices, **⊗** denotes the matrix Kronecker product), and estimate the linear model **y = Uη + ϵ** by least squares, where **U = [Z, X]** is the *nm* × (*m* + *p*) combined matrix and **η = (μ** ^*'*^, **ν** ^*'*^)^*′*^ is the combined vector of intercepts and slopes; compute the *m* × 1 residual vectors {**r**_*i*_, *i* = 1, …, *n*}. (1) Apply the *K* ‐means algorithm to {**r**_*i*_} to get index sets {*C*_1_, …, *C*_*K*_}. (2) Build an (*nm*) × (*Km*) matrix **Z = E ⊗ I**_*m*_, where **E** is the *n* × *K* matrix such that *E*_*ik*_ = 1 if *i* ∈ *C*_*k*_ and *E*_*ik*_ = 0 otherwise; estimate the linear model **y = Uη + ϵ,** compute the residual vectors **r**_*i*_, and return to step (1). Iterate until criterion (10) stops decreasing.

**Figure 10:**
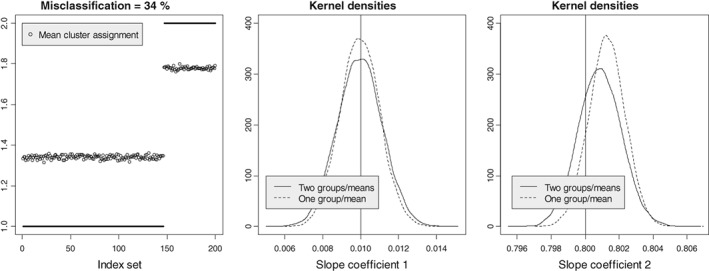
Statistical simulations for a clusterwise regression, N_sim_ = 10 000

EXAMPLE 5 *Statistical simulations for clusterwise regression*. An advantage of a statistical model for classification is that one can generate data and study the statistical properties of clustering through simulations. Consider the following regression problem with a three‐dimensional dependent variable (*m* = 3) and 2 slope coefficients (*p* = 2). Two groups of observations (*K* = 2)—146 observations from the first group (mean vector **μ**_1_) and 54 from the second (mean vector **μ**_2_)—have to be identified along with estimation of the 2 slope coefficients (*n* = 200). Let *σ* = 0.75, with the Mahalanobis distance between group‐specific intercepts *D* = ||**μ**_1_ − **μ**_2_||/*σ* = 1.6. How well can the observations be classified into 2 groups, and what is the statistical distribution of the slope coefficients? In particular, we want to understand the impact of grouping on the distribution of the slope coefficients. The results of 10 000 simulations with the data generated according to model (9) are presented in Figure 10. For each simulated dataset, the repeated *K*‐means algorithm was applied (typically it took about 4‐5 iterations to converge), and the index sets *C*_*k*_ and slope coefficients were estimated. The slope coefficients were also estimated under the assumption that there were no clusters using the standard linear model for a benchmark comparison. The left plot in Figure 10 shows the results of clustering in 10 000 experiments; the fact that the first 146 observations belong to cluster 1 and the remaining 54 observations belong to cluster 2 (the ground truth) is shown with the horizontal black lines. The average cluster assignment for each *i* is depicted with a circle. Approximately 34% of observations were wrongly assigned to another cluster (interestingly, each *i* has almost the same misclassification error). The distribution of 10 000 slope coefficients is shown in the plots at right. The solid line depicts the Gaussian kernel density estimate from the clusterwise regression, and the dotted line depicts the density of the coefficients when the presence of clusters is ignored; the vertical line indicates the true value of the coefficient. In both cases, the no‐cluster distribution (1 group/mean) is tighter with an underestimated standard deviation. The estimates of the second slope are positively biased in both methods; however, the two‐group model has a smaller bias.

## 6 CLUSTERING OF MULTILEVEL DATA

Traditional cluster algorithms work under the assumption of data homogeneity. Sometimes, the data to classify have a multilevel structure; for example, we may want to classify subjects for whom repeated measurements (replicates) are available. Such data will be referred to as multilevel data. The following example illustrates the concept.

**Figure 11:**
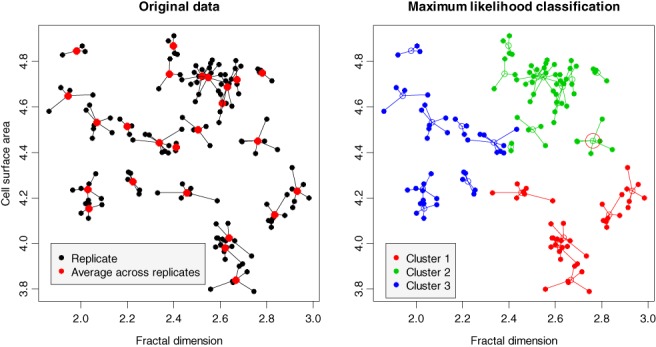
Right: AFM cell imaging for cervical cancer discrimination. The data consist of 2 image characteristics, fractal dimension and cell surface area, of 138 cells from the cervix of 25 women (2‐10 cells in each sample). Black dots are connected to averages (red dots). Left: The result of the ML classification. The large circle indicates the misclassified woman

EXAMPLE 6 *Atomic force microscopy (AFM) for cervical cancer detection*. Several studies report that AFM imaging can discriminate normal and cancer cervical cells using physical characteristics of the cell surface 14, 15. Figure 11 depicts a typical distribution of 2 cell AFM image characteristics, namely fractal dimension and cell surface area. The original data (the left plot) represents cell samples from a pap smear exam from *n* = 25 women; each exam sample contained 2 to 10 cells (black filled circles); the red circle is the average across replicates (red filled circle). We use segments to connect replicates to averages for a better hierarchy visualization; overall there are 138 pairs of observations. The ground truth is known: there are 3 types of samples: (1) normal cells (10 women), (2) squamous cell carcinoma (7 women), and (3) adenocarcinoma (8 women). Can the *K* ‐means algorithm identify the type of the woman's cervix cells in an unsupervised fashion? Two approaches are obvious: (1) use cells as the measurement unit and apply the *K*‐means to all *n* = 138 two‐dimensional vectors, or (2) apply the *K*‐means to averages over replicates (red circles), *n* = 25. The first approach may lead to confusion because replicates from 1 woman may be assigned to different clusters. The second approach treats the averages equal, but in fact one has to take into account the number of averaged replicates. The following statistical model takes into account the hierarchy of the data by recognizing the difference between the variation of image characteristics within each woman and between women (women heterogeneity).

The statistical model for classification with replicates takes the form of the variance components model 21, 35, but the groups are not known. As before, **x** indicates the vector of observations, but now it has 2 indices: The first index *i* indicates the observation to be classified (the woman in the AFM example), and the second index *j* indicates a replicate (there are *p*_*i*_ replicates for woman *i*). The statistical model can be viewed as a simple mixed model 11

(11) $$x_{\mathrm{ij}}=\mu_{k}+b_{i}+\epsilon_{\mathrm{ij}},\;j=1,2,\ldots ,p_{i},\;i\in C_{k},$$

where

$$b_{i}∼N\left(0,\sigma^{2}\tau^{2}I_{m}\right),\;\epsilon_{\mathrm{ij}}∼N\left(0,\sigma^{2}I_{m}\right)$$

are random effects representing intra‐individual variation. Note that in the traditional mixed model, the clusters specified by the index sets are known; here we want to estimate them along with the means and variance parameters. In the AFM example, parameter *τ* ^2^ reflects the heterogeneity among women, and it is expected that *τ* ^2^ > 1, reflecting a commonly observed biological phenomenon: namely the variation between women is larger than the variation within woman. The total variance of **x**_*ij*_ is *σ* ^2^ + *σ* ^2^ *τ* ^2^ = *σ* ^2^(1 + *τ* ^2^), the sum of the variation across replicates of the same woman (*σ* ^2^) and the variation between women (*σ* ^2^ *τ* ^2^). This model implies that replicates corresponding to the same *i* correlate with the correlation coefficient *ρ* = *τ* ^2^/(1 + *τ* ^2^). The following theorem lists the facts about the ML classification of the multilevel data specified by model (11).

THEOREM 1 (a) If the number of replicates is the same (*p*_*i*_ = *p*), the maximum likelihood hard classification is achieved by the *K*‐means algorithm applied to the averages, ${\tilde{x}}_{i}=p^{-1}\sum_{j=1}^{p}x_{\mathrm{ij}}.$ (b) If the number of replicates is different, the maximum likelihood is equivalent to minimizing

(12) $$N\ln\left(S_{0}+\sum_{k=1}^{K}\sum_{i\in C_{k}}\frac{p_{i}}{1+p_{i}\tau^{2}}{\left\|\;{\tilde{x}}_{i}-\mu_{k}\right\|}^{2}\right)+m\sum_{i=1}^{n}\ln\left(1+p_{i}\tau^{2}\right),$$

over *τ*, **μ**_*k*_, and {*C*_*k*_, *k* = 1, …, *K*}, where $N=\sum_{i=1}^{n}p_{i}$ and

$$S_{0}=\sum_{i=1}^{n}\sum_{j=1}^{p_{i}}{\left\|x_{\mathrm{ij}}-{\tilde{x}}_{i}\right\|}^{2}.$$

(c) Minimization of (12) can be accomplished by alternating between the weighted *K*‐means algorithm using $\left\{{\tilde{x}}_{i}\right\}$ with weights *w*_*i*_ = *p*_*i*_/(1 + *p*_*i*_ *τ* ^2^) when *τ* ^2^ is held fixed, and the fix‐point algorithm for *τ* when **μ**_*k*_ and {*C*_*k*_} are held fixed:

(13) $$\tau_{t+1}^{2}\;=\;\tau_{t}^{2}\frac{N}{m}\frac{\sum_{i=1}^{n}\frac{h_{i}p_{i}}{{\left(1+p_{i}\tau^{2}\right)}^{2}}}{\left(\;,\sum_{i=1}^{n}\frac{p_{i}}{1+p_{i}\tau^{2}},\;\right)\left(\;,S_{0}\;+\;\sum_{i=1}^{n}\frac{h_{i}}{1+p_{i}\tau^{2}},\;\right)},\;t=0,1,\ldots ,$$

where $h_{i}=p_{i}{\left\|\;{\tilde{x}}_{i}-\mu_{k}\right\|}^{2},$ starting from

(14) $$\tau_{0}^{2}=\frac{N\sum_{i=1}^{n}h_{i}/p_{i}}{S_{0}\mathrm{mn}}.$$

(d) When *τ* ^2^ = 0, minimization of (12) turns into the weighted *K*‐means algorithm for $\left\{{\tilde{x}}_{i}\right\}$ with weights *w*_*i*_ = *p*_*i*_.

See the Appendix for the proof.

The fact that the ML estimation with an equal number of replicates reduces to the *K*‐means is understandable because then averages have the same variance and therefore can be treated equally. It is easy to prove that fix‐point iterations produce a positive solution if

(15) $$\sum_{i=1}^{n}h_{i}p_{i}>m\left(S_{0}+\sum_{i=1}^{n}h_{i}\right)$$

and otherwise *τ* ^2^ = 0. Indeed, consider the right‐hand side of expression (13) as a function of *τ* ^2^. This function approaches (14) when *τ*^2^ → ∞ . The solution is positive if the derivative of this function, evaluated at *τ* ^2^ = 0, is greater than 1—it is easy to see that this holds under the inequality (15).

EXAMPLE 7 *AFM cell imaging (continued)*. We apply the ML for hard classification to AFM cervical cell images using 2 the characteristics shown in the left plot of Figure 11. The R function cclust of package flexclust is used to run the weighted *K*‐means algorithm when *τ* ^2^ is held fixed. The results of classification are shown in the right plot of Figure 11. Only 1 woman, indicated with a large red circle, is misclassified. She belongs to Cluster 1 (adenocarcinoma), but the ML hard classification put her into Cluster 2 (squamous cell carcinoma).

## 7 CONCLUSIONS

Typical cluster analysis stops after classification is complete. For the next‐generation *K*‐means algorithm, the work is about to start: What is the confidence interval for the mean vector, and how well are the index sets *C*_*k*_ estimated? How to test that clusters exist? What is the number of clusters and what is their distribution of its estimate? What are statistical properties of clusterwise regression coefficients? These questions cannot be answered based on the standard algorithm‐driven paradigm. The only way to study the properties of the classification is to use a model‐based *K*‐means algorithm. This model was proposed by Banfield and Raftery more than 20 years ago, but little has been done since.

We have developed new directions and extensions to the statistical model‐based *K*‐means algorithm which turns into the ML estimation. But it is too early to claim victory: The hard classification problem does not fall into the track of the well‐established statistical theory because the number of parameters grows with *n* and the index sets are discrete. Special statistical methods, married with combinatorics, are required, and simulations here will be very helpful.

The hard classification problem, and particularly finding the optimal partition set, may have several local solutions. Development of the global minimum criteria, following the route of continuous optimization 10, is a matter of future work. We strongly recommend the use of at least 10 starting points in the *K*‐means algorithm to ensure that the global minimum has been achieved (kmeans[…,nstart = 10 ,…] in R).

Obviously, 1 paper cannot solve multiple problems emerging in connection with extension of the *K*‐means algorithm. However, we hope that our work will stimulate interest in further development of hard classification algorithms and deeper understanding of their statistical properties.

## APPENDIX: PROOF OF THEOREM

(a) Stack the replicates of the *i*th observation vector from cluster *k* to form a (*mp*) × 1 vector $X_{i}={\left(x_{i1}^{\prime },\ldots ,x_{\mathrm{ip}}^{\prime }\right)}^{\prime }$. Its distribution is multivariate normal, $X_{i}∼N\left(1_{p}⊗\mu_{k},\sigma^{2}N\right),$ where **1**_*p*_ is the *p* × 1 vector of 1's and

$$N=I_{\mathrm{mp}}+\tau^{2}1_{p}1_{p}^{\prime }⊗I_{m}$$

is the (*mp*) × (*mp*) symmetric matrix. Using formulas of matrix algebra, one can show that the determinant and the matrix inverse can be derived in closed forms as follows:

$$\left|N\right|={\left(1+p\tau^{2}\right)}^{m},\;N^{-1}=I_{\mathrm{mp}}-\frac{\tau^{2}}{1+p\tau^{2}}1_{p}1_{p}^{\prime }⊗I_{m}.$$

Therefore, the twice‐negative log‐likelihood function for the *i*th observation from cluster *k*, up to a constant term, can be written as

$$\begin{array}{l} l_{i}\left(\mu_{K},\sigma^{2},\tau^{2}\right)=\mathrm{mp}\ln\sigma^{2}+m\ln\left(1+p\tau^{2}\right)\\ +\frac{1}{\sigma^{2}}{\left(X_{i}-1_{p}⊗\mu_{k}\right)}^{\prime }\left(I_{\mathrm{mp}}-\frac{\tau^{2}}{1+p\tau^{2}}1_{p}1_{p}^{\prime }⊗I_{m}\right)\\ \left(X_{i}-1_{p}⊗\mu_{k}\right). \end{array}$$

After some matrix algebra, we obtain

$$\begin{array}{cc} & {\left(X_{i}-1_{p}⊗\mu_{k}\right)}^{\prime }\left(I_{\mathrm{mp}}-\frac{\tau^{2}}{1+p\tau^{2}}1_{p}1_{p}^{\prime }⊗I_{m}\right)\left(X_{i}-1_{p}⊗\mu_{k}\right)\\ & \;=S_{i}-\frac{\tau^{2}}{1+p\tau^{2}}M_{i}, \end{array}$$

where

$$S_{i}=\sum_{j=1}^{p}{\left\|x_{\mathrm{ij}}-\mu_{k}\right\|}^{2},\;M_{i}={\left\|\sum_{j=1}^{p}\left(x_{\mathrm{ij}}-\mu_{k}\right)\right\|}^{2}$$

to shorten the notation. After these simplifications, the twice‐negative log‐likelihood function to be minimized takes the form

(A1) $$\mathrm{nmp}\ln\sigma^{2}+\mathrm{nm}\ln\left(1+p\tau^{2}\right)+\frac{1}{\sigma^{2}}\sum_{k=1}^{K}\sum_{i\in C_{k}}\left(S_{i}-\frac{\tau^{2}}{1+p\tau^{2}}M_{i}\right).$$

The minimum of this function over *σ* ^2^ is attained at

$$\sigma^{2}=\frac{1}{\mathrm{nmp}}\sum_{k=1}^{K}\sum_{i\in C_{k}}\left(S_{i}-\frac{\tau^{2}}{1+p\tau^{2}}M_{i}\right).$$

Plugging this back into expression (A1) leads to the minimization of

(A2) $$p\ln\sum_{k=1}^{K}\sum_{i\in C_{k}}\left(S_{i}-\frac{\tau^{2}}{1+p\tau^{2}}M_{i}\right)+\ln\left(1+p\tau^{2}\right).$$

Differentiating this function with respect to *τ* ^2^ and setting it to zero leads to the minimum, again up to a constant:

(A3) $$\begin{array}{l} \left(p-1\right)\ln\sum_{k=1}^{K}\sum_{i\in C_{k}}\left(\sum_{j=1}^{p}{\left\|x_{\mathrm{ij}}-\mu_{k}\right\|}^{2}-p^{-1}{\left\|\sum_{j=1}^{p}\left(x_{\mathrm{ij}}-\mu_{k}\right)\right\|}^{2}\right)\\ +\ln\sum_{k=1}^{K}\sum_{i\in C_{k}}{\left\|\sum_{j=1}^{p}\left(x_{\mathrm{ij}}-\mu_{k}\right)\right\|}^{2}. \end{array}$$

But

$$\sum_{j=1}^{p}{\left\|x_{\mathrm{ij}}-\mu_{k}\right\|}^{2}-p^{-1}{\left\|\sum_{j=1}^{p}\left(x_{\mathrm{ij}}-\mu_{k}\right)\right\|}^{2}=\sum_{j=1}^{p}{\left\|x_{\mathrm{ij}}-{\tilde{x}}_{i}\right\|}^{2},$$

where ${\tilde{x}}_{i}=p^{-1}\sum_{j=1}^{p}x_{\mathrm{ij}},$ the average of the replicates. The first term in (A3)

$$\left(p-1\right)\ln\sum_{k=1}^{K}\sum_{i\in C_{k}}\sum_{j=1}^{p}{\left\|x_{\mathrm{ij}}-{\tilde{x}}_{i}\right\|}^{2},$$

does not depend on {*C*_*k*_}. The minimum of the second term over **μ**_*k*_ is attained at

(A4) $${\bar{x}}_{k}=\frac{1}{{\mathrm{pn}}_{k}}\sum_{i\in C_{k}}\sum_{j=1}^{p}x_{\mathrm{ij}}=\frac{1}{n_{k}}\sum_{i\in C_{k}}{\tilde{x}}_{i},$$

where *n*_*k*_ is the number of elements in the *k*th cluster. Since $\sum_{j=1}^{p}\left(x_{\mathrm{ij}}-{\bar{x}}_{k}\right)=p\left({\tilde{x}}_{i}-{\bar{x}}_{k}\right),$ the minimization of (A3) is equivalent to the minimization of

$$\sum_{k=1}^{K}\sum_{i\in C_{k}}{\left\|\;{\tilde{x}}_{i}-{\bar{x}}_{k}\right\|}^{2}$$

as a result of the second equality in (A4). Finally, we conclude that the ML hard classification is achieved by the *K*‐means algorithm applied to ${\tilde{x}}_{i}.$

(b) We proceed as in the previous case, but now vector and matrix dimensions may vary with *i*. For example, $X_{i}∼N\left(1_{p_{i}}⊗\mu_{k},\sigma^{2}N_{i}\right)$ and $N_{i}=I_{{\mathrm{mp}}_{i}}+\tau^{2}1_{p_{i}}1_{p_{i}}^{\prime }⊗I_{m}.$ Using the previous formulas for the determinant and inverse, we arrive at the analog of (A1):

(A5) $$\mathrm{mN}\ln\sigma^{2}+m\;\sum_{i=1}^{n}\ln\left(1+p_{i}\tau^{2}\right)+\frac{1}{\sigma^{2}}\sum_{k=1}^{K}\sum_{i\in C_{k}}\left(\;,S_{i}-\frac{\tau^{2}}{1+p_{i}\tau^{2}}M_{i},\;\right),$$

where $N=\sum_{i=1}^{n}p_{i}$ is the total number of vectors to classify and

$$S_{i}=\sum_{j=1}^{p_{i}}{\left\|x_{\mathrm{ij}}-\mu_{k}\right\|}^{2},\;M_{i}=p_{i}^{2}{\left\|\;{\tilde{x}}_{i}-\mu_{k}\right\|}^{2}.$$

Again, by eliminating *σ* ^2^, the minimization of (A5) turns into the minimization of

(A6) $$N\ln\sum_{k=1}^{K}\sum_{i\in C_{k}}\left(S_{i}-\frac{\tau^{2}}{1+p_{i}\tau^{2}}M_{i}\right)+m\sum_{i=1}^{n}\ln\left(1+p_{i}\tau^{2}\right).$$

Express *S*_*i*_ through ${\tilde{x}}_{i}$ using the elementary identity ||**a** + **b**||^2^ = ||**a**||^2^ + 2**a**^′^**b** + ||**b**||^2^ to obtain

$$\begin{array}{l} S_{i}=\sum_{j=1}^{p_{i}}{\left\|\left(x_{\mathrm{ij}}-{\tilde{x}}_{i}\right)+\left({\tilde{x}}_{i}-\mu_{k}\right)\right\|}^{2}\\ =\sum_{j=1}^{p_{i}}{\left\|(x_{\mathrm{ij}}-{\tilde{x}}_{i}\right\|}^{2}+2\sum_{j=1}^{p_{i}}{\left(x_{\mathrm{ij}}-{\tilde{x}}_{i}\right)}^{\prime }\left({\tilde{x}}_{i}-\mu_{k}\right)+\sum_{j=1}^{p_{i}}{\left\|{\tilde{x}}_{i}-\mu_{k}\right\|}^{2}\\ =\sum_{j=1}^{p_{i}}{\left\|(x_{\mathrm{ij}}-{\tilde{x}}_{i}\right\|}^{2}+p_{i}{\left\|{\tilde{x}}_{i}-\mu_{k}\right\|}^{2} \end{array}$$

since $\sum_{j=1}^{p_{i}}\left(x_{\mathrm{ij}}-x_{i}\right)=0.$ Further, after simplifying

$$S_{i}-\frac{\tau^{2}}{1+p_{i}\tau^{2}}M_{i}=S_{0}+\frac{p_{i}}{1+p_{i}\tau^{2}}{\left\|{\tilde{x}}_{i}-\mu_{k}\right\|}^{2},$$

the minimization of function (A6) turns into the minimization of

$$N\ln\left(S_{0}+\sum_{k=1}^{K}\sum_{i\in C_{k}}\frac{p_{i}}{1+p_{i}\tau^{2}}{\left\|{\tilde{x}}_{i}-\mu_{k}\right\|}^{2}\right)+m\sum_{i=1}^{n}\ln\left(1+p_{i}\tau^{2}\right),$$

where

$$S_{0}=\sum_{i=1}^{n}\sum_{j=1}^{p_{i}}{\left\|(x_{\mathrm{ij}}-{\tilde{x}}_{i}\right\|}^{2}.$$

This means that the ML estimation is equivalent to the minimization of (12).

(c) When **μ**_*k*_ and {*C*_*k*_} are obtained from the weighted *K*‐means algorithm and held fixed, the maximum of (12) occurs when the derivative with respect to *τ* ^2^ is zero or, equivalently, when the following equation holds:

$$\sum_{i=1}^{n}\frac{h_{i}p_{i}}{{\left(1+p_{i}\tau^{2}\right)}^{2}}=\frac{m}{N}\left(\sum_{i=1}^{n}\frac{p_{i}}{1+p_{i}\tau^{2}}\right)\left(S_{0}+\sum_{i=1}^{n}\frac{h_{i}}{1+p_{i}\tau^{2}}\right),$$

where $h_{i}=p_{i}{\left\|{\tilde{x}}_{i}-\mu_{k}\right\|}^{2}.$ Rewrite the above equation as

$$\sum_{i=1}^{n}\frac{h_{i}p_{i}\tau^{4}}{{\left(1+p_{i}\tau^{2}\right)}^{2}}=\tau^{2}\frac{m}{N}\left(\;,\sum_{i=1}^{n}\frac{\tau^{2}p_{i}}{1+p_{i}\tau^{2}},\;\right)\left(\;,S_{0}+\sum_{i=1}^{n}\frac{h_{i}}{1+p_{i}\tau^{2}},\;\right)$$

and equivalently

$$\tau^{2}=\frac{N}{m}\frac{\sum_{i=1}^{n}\frac{h_{i}p_{i}\tau^{4}}{{\left(1+p_{i}\tau^{2}\right)}^{2}}}{\left(\sum_{i=1}^{n}\frac{\tau^{2}p_{i}}{1+p_{i}\tau^{2}}\right)\left(S_{0}+\sum_{i=1}^{n}\frac{h_{i}}{1+p_{i}\tau^{2}}\right)},$$

which gives rise to the fix‐point iterations (13) starting from

$$\tau_{0}^{2}=\frac{N\sum_{i=1}^{n}h_{i}/p_{i}}{S_{0}\mathrm{mn}}.$$

The alternation between the weighted *K*‐means algorithm and the fixed‐point iterations for *τ* ^2^ are continued until (12) does not decrease by a small *ϵ*.

(d) When *τ* ^2^ = 0, as follows from (12), the ML turns into the minimization of

$$\sum_{k=1}^{K}\sum_{i\in C_{k}}p_{i}{\left\|{\tilde{x}}_{i}-\mu_{k}\right\|}^{2},$$

which is the weighted *K*‐means algorithm.
